# Biomarkers and severe asthma: a critical appraisal

**DOI:** 10.1186/s12948-015-0027-7

**Published:** 2015-10-01

**Authors:** Alessandra Chiappori, Laura De Ferrari, Chiara Folli, Pierluigi Mauri, Anna Maria Riccio, Giorgio Walter Canonica

**Affiliations:** DIMI-Department of Internal Medicine, Respiratory Diseases and Allergy Clinic, University of Genoa, IRCCS AOU S.Martino-IST, Genoa, Italy; Institute for Biomedical Technologies, CNR, Segrate, Milan, Italy

**Keywords:** Severe asthma, Biomarkers, Exhaled nitric oxide, Periostin, Eosinophil, Galectin, Allergic inflammation, Neutrophil, Monoclonal antibodies

## Abstract

Severe asthma (SA) is a clinically and etiologically heterogeneous respiratory disease which affects among 5–10 % of asthmatic patients. Despite high-dose therapy, a large patients percentage is not fully controlled and has a poor quality of life. In this review, we describe the biomarkers actually known in scientific literature and used in clinical practice for SA assessment and management: neutrophils, eosinophils, periostin, fractional exhaled nitric oxide, exhaled breath condensate and galectins. Moreover, we give an overview on clinical and biological features characterizing severe asthma, paying special attention to the potential use of these ones as reliable markers. We finally underline the need to define different biomarkers panels to select patients affected by severe asthma for specific and personalized therapeutic approach.

## Background

Severe asthma (SA) is defined as ‘asthma which requires treatment with high dose inhaled corticosteroids (ICS) plus a second controller (and/or systemic corticosteroids) to prevent it from becoming ‘uncontrolled’ or which remains ‘uncontrolled’ despite this therapy’. This is the European Respiratory Society (ERS)/American Thoracic Society (ATS) definition, utilized across developed countries with general access to inhaled corticosteroid therapy [1, 2]. It is recognized that up to 50 % of patients are not well controlled and 5–10 % of patients suffer from a particularly severe disease that is often refractory to usual treatment [3, 4].

Evidence of any one of the four criteria below, while on current high-dose therapy, identifies the patient as having ‘‘severe asthma’’:

1. Poor symptoms control.
2. Frequent severe exacerbations, defined as two or more bursts of systemic corticosteroids in the previous year.
3. Serious exacerbations, defined as at least one hospitalization, intensive care unit stay or mechanical ventilation in the previous year.
4. Airflow limitation, i.e. forced expiratory volume in 1 s (FEV1), 80 % predicted (in the presence of reduced FEV1/forced vital capacity (FVC) defined as less than the lower limit of normal) following a withhold of both short- and long-acting bronchodilators.

Asthma, and severe asthma in particular, are increasingly considered as heterogeneous diseases, which may respond similarly to therapies. Recently, studies are beginning to identify different phenotypes defined by characteristic clinical manifestations, pathophysiological mechanisms and biomarkers [5].

The first studies performed in asthmatics distinguished subtypes based on inflammatory patterns obtained from bronchoalveolar lavage and endobronchial biopsies. Identifying patients with corticosteroid-naive, mild asthma who exhibited a T-helper (Th)2/Type 2 molecular signature in their epithelial cells, Woodruff et al., began the concept of molecular phenotyping, in which molecular pathways are linked to clinical and physiological characteristics [6].

A more detailed immunopathobiological picture of human SA is therefore emerging as the context of the heterogeneity of the disease, in relation to both inflammation and structural changes [7].

The identification of asthma phenotypes has given a boost to the search for biomarkers to help classifying patients, targeting therapies and predicting different pathological evolution mechanisms of the disease with strong benefits for the affected patients [8].

An ideal biomarker is easy to collect and measure, not invasive nor expensive, and can be used to identify either clinical or treatment response phenotypes, evaluate changes in disease activity, or confirm a diagnosis. This prospect provides the impetus for the research for reliable markers in SA, which are actually under intense study and are hereunder analyzed. In this review we focus the importance and the role of different known and potential biomarkers useful to define the physiopathological and clinical profiles of patients affected by severe asthma.

### Neutrophils

Severe asthma is often characterized by neutrophilic inflammation, both in the presence or absence of Th2-induced eosinophilic inflammation [9, 10]. However, the functional role of these cells in disease progression remains unclear. Sputum neutrophil percentages are highly reproducible in patients with moderate to severe asthma, and can be used to assess novel anti-inflammatory therapies: targeting neutrophils has been suggested as a therapeutic option for these patients [11, 12].

Neutrophils recruitment can be mediated by Th17 cells, which are thought to have a role in asthma pathogenesis, especially in patients who do not respond to glucocorticoid therapy and show a decreased improvement in FEV1 and airway hyperresponsiveness (AHR) following treatment [13, 14]. Moreover, it has been demonstrated that, in addition to the specific antigenic components, pollens contain several intrinsic factors able to promote innate immune responses; among them, nicotinamide adenine dinucleotide phosphate oxidase (NADPH) induces the generation of reactive oxygen species (ROS) [15]. This oxidative insult induces an early wave of neutrophil recruitment in the airways and increases significantly ROS-generating activity of these cells [16]. It has been also evidenced that pollen can induce CXCL chemokine synthesis and that neutrophils can be recruited into the airways in a CXCR2-dependent manner [17].

The receptor for advanced glycation end-products (RAGE) is a pattern recognition receptor involved in the response to injury, infection and inflammation. Perturbations in the RAGE and its soluble forms (sRAGE) balance might be linked to neutrophilic airway inflammation in chronic airways disease [18]. A recent work from Sukkar et al. demonstrated a deficiency in lung and systemic sRAGE, in asthmatic and chronic obstructive pulmonary disease (COPD) patients with neutrophilic airway inflammation. sRAGE might be degraded by neutrophil-derived proteolytic enzymes in subjects with airway neutrophilia and might be identified as a potential biomarker for prognosis or patient management [19].

The metalloproteinase domain 8 (ADAM8) seems also to be involved in facilitating neutrophils migration into tissues [20]. It is highly expressed in bronchial biopsies from moderate and severe asthmatics and its recruitment of both eosinophils and neutrophils into airways tissue suggests a significant role in the pathogenesis of asthma [21–24].

Future therapeutic targets directed at the above mentioned proteins might significantly attenuate asthma symptoms by reducing inflammatory cells, primarily neutrophils, and having few adverse physiological consequences.

### Eosinophils

Asthma is also histologically characterized by recruitment of eosinophils into the large airway wall and lumen, along with mucus plugging and epithelium denudation. Being associated with increased transforming growth factor β (TGF-β) expression and reticular basement membrane (RBM) thickness, the amount of these cells might be congruent with symptoms severity, worsened lung function, and near-fatal events [25–27].

The identification of eosinophilic inflammation is of a certain importance due to the severity of attacks which occur to these patients and to the existence of drugs, such as prednisolone, omalizumab and new biological agents, particularly active on this pattern [28].

It is known that Type-2 inflammatory pathways are involved in asthma, and many studies suggest that about 50 % of SA patients present Type-2 inflammation, as measured by eosinophilia or high levels of fractional exhaled nitric oxide (FeNO) [29, 30]. Miranda et al. distinguished early-onset severe asthma patients, characterized by allergen sensitivity, allergic symptoms, eosinophilia and higher serum immunoglobulin E (IgE) levels, and late-onset severe asthma subjects, with lower lung function than early-onset ones, despite a shorter duration of illness and significantly more symptoms if presenting persistent eosinophils at onset [27].

The need to block IgE binding to inflammatory cells and the consequent mediators release cascade concurred to the development of the unique monoclonal antibody approved for patients with severe allergic asthma: omalizumab, a recombinant humanized murine antibody against IgE antibodies. A recent study from our group investigated the effect of long-term anti-IgE treatment on the thickening of the RBM and eosinophil infiltration in bronchial biopsies from patients with severe persistent allergic asthma. Our results showed that a substantial proportion of severe asthmatics reduced the original bronchial RBM thickness and eosinophil infiltration after one-year treatment with anti-IgE, thus emphasizing the possible role of omalizumab in affecting airway remodeling in severe persistent allergic asthma [31]. Up to now, many studies have shown how the anti-IgE treatment in SA patients was effective in modulating airways remodeling and inflammation, as well as in improving lung functions and quality of life (Table 1).

**Table 1:** Studies on clinical effectiveness of omalizumab in patients with severe asthma

Mediators and parameters	Biologic sample/procedure	Patients	Effects of omalizumab treatment	References
ET-1 FeNO ECP Eosinophils count FEV1	EBC EB Blood Blood Spirometry	19 severe asthmatics 9/19 omalizumab (+)	↓ ↓ ↓ ↓ ↑	[32]
RANTES/CCL5 FeNO ECP Eosinophils count	EBC EB Blood Blood	19 severe asthmatics 9/19 omalizumab (+)	↓ ↓ ↓ ↓	[33]
Eosinophils count	Blood	13 severe sthmatics 13/13 omalizumab (+)	↓	[34]
Quality of life PEF Unscheduled visits Exacerbations FeNO CalvNO Eosinophils count Airway-wall thickness	Questionnaire Spirometry Clinical data Clinical data EB EB Sputum CT	26 severe asthmatics 26/26 omalizumab (+)	↑ ↑ ↓ ↓ ↓ ↓ ↓ ↓	[35]
Exacerbations Systemic steroids ACT score	Clinical data Clinical data Questionnaire	22 severe asthmatics 22/22 omalizumab (+)	↓ ↓ ↑	[36]
Airway-wall thickness Eosinophils count	Bronchial biopsies	11 severe asthmatics 11/11 omalizumab (+)	↓ ↓	[31]
Bronchial smooth muscle proteins	Bronchial biopsies	8 severe astmatics 8/8 omalizumab (+)	↓	[37]

*ET-1* endothelin-1, *FeNO* fractional exhaled nitric oxide, *ECP* eosinophil cationic protein, *FEV1* forced expired volume in 1 s, *EB* exhaled breath, *EBC* exhaled breath condensate, *RANTES/CCL5* regulated on activation, normal T cell expressed and secreted/chemokine (C–C motif) ligand-5, *PEF* peak expiratory flow, *CalvNO* estimated alveolar nitric oxide concentration, *CT* computerized tomography, *ACT* asthma control test.

Eosinophils count in induced sputum has long been the method of choice to evaluate eosinophilic lung inflammation and seems to be a reliable biomarker of airway inflammation as well as useful in adjusting corticosteroid treatments in asthma [38–40].

Newby et al. and McGrath et al. by using sputum, identified phenotypes of SA enriched for eosinophilic airway inflammation that might respond to therapies directed toward Th2 immune pathways [41, 42].

Recently, the dose ranging efficacy and safety with mepolizumab (DREAM) study correlated blood eosinophils levels, but not sputum eosinophilia, with response to mepolizumab [43]. These data are supported by many authors who evidenced that sputum eosinophils can not predict treatment response and that increased blood eosinophils are associated with higher risk for exacerbations, maybe owing to interleukin (IL)-5 levels [44, 45]. Following anti-IgE treatment, some studies found decreased blood eosinophils counts [46]. In the EXTRA study, Hanania and colleagues demonstrated that omalizumab efficacy was strongly related to the presence of airways eosinophilic inflammation and more accurately predicted by FeNO and serum periostin rather than IgE levels [47].

It is currently hypothesized that the use of blood eosinophils as biomarkers could help to personalize asthma management in patients with severe allergic asthma.

Active eosinophils recruitment is predominantly exerted by proteins secreted by epithelia, the most potent chemoattractant for these cells being eotaxin-1 (CCL11), that account for 80 % of TGF-ß expression in asthma [48–50]. A recent work evaluated CCL11 levels in bronchoalveolar lavage fluid (BALF), exhaled breath condensate (EBC), blood and sputum and evidenced a correlation between this protein in induced sputum and asthma severity [51].

It has also been hypothesized that asthma severity might be linked to a relationship between TGF-ß expression and the presence of submucosal eosinophils. It was evidenced that in bronchial biopsies the majority of eosinophils is TGF-ß1-mRNA positive [25, 52], with a higher extent in SA. Moreover, TGF-ß2 isoforms are expressed by eosinophils in severe allergic asthma where this cytokine promotes fibrotic responses and regulates mucin production [53, 54].

Although still in study and source of debate, the persistent eosinophilic phenotype in adults might be a real candidate for specific therapies thus potentially interfering with the natural history of SA with high exacerbation rates.

### Fractional exhaled nitric oxide

Epithelial inducible nitric oxide synthase (NOS) has been shown to be the main determinant of FeNO levels in the respiratory tract [55] due to increased nitric oxide (NO) production by activated bronchial epithelial cells in response to pro-inflammatory stimuli [56]. It is postulated that the decrease in FeNO values seen after steroid treatment might be due to an inhibitory effect of these drugs on inducible NOS activity [55], therefore the measurement of NO concentration in exhaled breath has been standardized for clinical use. It is a quantitative, non-invasive, simple, and safe method to assess airway inflammation, to monitor responsiveness to ICS therapy in adults [57] and to predict asthma control status in childhood [58]. Recently, a retrospective study was performed on 416 asthmatic patients on combined therapy (long-acting β2 agonist and ICS). The authors assessed the correlation between FEV1 and FeNO to ascertain the correct use of FeNO measurement in different asthma phenotypes with regard to disease control, severity, allergy, comorbidity, obesity, age and smoking status. No correlation was found between FeNO levels and asthma severity but it was shown a link to other parameters such as age, gender, history of emergency room visits and atopy [59]. Moreover, cross-sectional study on 100 adult asthmatic patients showed that FeNO levels were correlated primary with asthma control rather than asthma severity, confirming FeNO as reliable biomarker in asthma management [60]. Peirsman et al. performed a randomized controlled trial on 99 children with persistent allergic asthma: patients outcomes were evaluated over a 52-week timeframe and, only in FeNO group, therapeutic approach was guided by FeNO measurements. Results demonstrated that FeNO evaluation diminished asthma exacerbations rate in associated with an increased leukotriene receptor antagonist use and ICS doses administration [61]. FeNO may also be used as predictor of omalizumab treatment efficacy. Hanania et al. evaluated FeNO, serum periostin and blood eosinophilia as potential biomarkers useful to evaluate effectiveness of anti-IgE treatment on 850 adult persistent SA patients. After 48 weeks of therapy, reduction of exacerbations rate was greater in high versus low subgroup for FeNO levels (53 vs 16 %) [47]. In the Inner-City Anti-IgE therapy of asthma trial, Busse et al. confirmed a clinical benefit from omalizumab treatment in children and adolescents [62]. In a post hoc analysis of the previous study, Sorkeness et al. found that FeNO, together with blood eosinophils and body mass index, can predict omalizumab response [63]. Moreover, preliminary data indicated that elevated FeNO may be indicative of anti-IL-13/IL-4 biological response [64]. However, Haldar et al. showed that FeNO seems to be less closely associated with a response to mepolizumab (anti IL-5) than blood eosinophils count [65].

In SA, the bronchial mucosa is markedly hyperplastic, the epithelium may be susceptible to destruction, flaking and detachment from the RBM. Therefore, FeNO levels can be under-representative [66]. The ERS/ATS Task Force has published a detailed guideline on definition, evaluation and treatment of SA. The Authors defined SA phenotypes giving specific recommendations for the use of diagnostic tools like measurement of FeNO to guide therapy. Elevated FeNO value is considered as a marker of Th2 inflammation and atopy but not an effective biomarker useful in SA management [1].

### Exhaled breath condensate

**Fig. 1:**
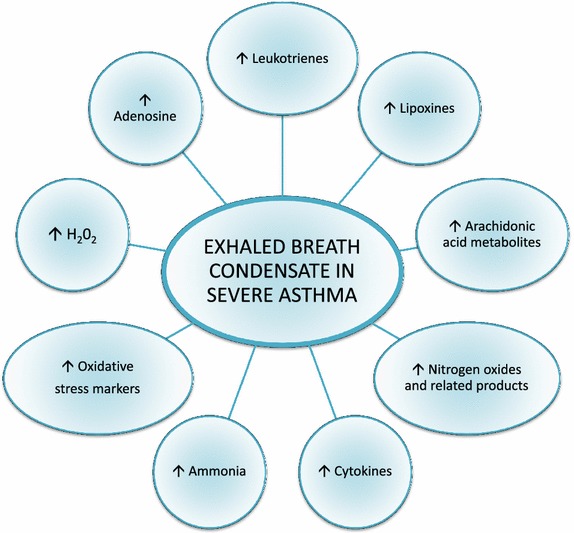
Changes in exhaled breath condensate mediators values in severe asthma.

Exhaled breath (EB) is mainly composed by aerosolized, non-volatile particles of airway lining fluid collected from the airways by airflow turbulence, water vapor condensation, inorganic (O_2_, N_2_), and organic (CO_2_) atmospheric volatile water-soluble gases, endogenous and exogenous volatile organic compounds [67]. EBC is the EB that has been condensed, typically by using a commercially available refrigerating device, according to ATS/ERS Guidelines [68]. It is used to investigate the composition of airway fluids and to achieve information about pulmonary alveoli, inflammation, nitrosative and oxidative stress in airways diseases such as COPD [69], asthma [70] and lung cancer [67]. Concerning SA, clinical studies identified chemical and biological characteristics that distinguish EBC of severe asthmatics from those obtained from healthy subjects and mild to moderate asthmatic patients (Fig. 1). Experimental data on EBC pH in SA are contrasting and there are still few studies about it [71, 72]. Eicosanoids as pro-inflammatory Leukotriene B4 (LTB4) and anti-inflammatory Lipoxin A4 (LXA4) are increased in asthmatics versus healthy subjects and LXA4/LTB4 ratio dramatically decreases in EBC in correlation with asthma severity [73]. Eotaxin-1 was evaluated in blood, EBC, sputum and BALF and was proposed as a tool for assessment of asthma severity [51]. High levels of nitrogen reactive species (NO, NO^2−^, NO^3−^) and endogenous ROS (superoxide, hydrogen peroxide, hydroxyl radical) provided evidence for pathologic oxidizing processes in asthma and are indicative of airways oxidative and nitrosative stress [74]. In SA, these pathologic features are exacerbated and correlated to asthma stability and corticosteroid therapy response [75]. Many studies have been published on the use of omics techniques in asthmatic children for the analysis of easy to be collected samples. Using liquid chromatography and mass spectrometry, significant differences useful to distinguish asthma severity between healthy and asthmatic children biochemical profiles were found in urine and in EBC [76–78]. Baraldi et al. proposed the use of metabolomic analysis or breathomic approach of EBC to characterize asthma phenotypes and personalize the therapeutic plan [79]. Fitzpatrick et al. found that SA children showed metabolic differences associated with oxidative stress-related pathways which may contribute to their corticosteroids refractory state [80]. General recommendations for EBC collection and exhaled biomarkers measurements are available in order to avoid a possible alteration in biomarker concentrations. Despite EBC collection remains a procedure potentially influenced by numerous endogenous and exogenous factors, in present and in future, EBC analysis can turn out as a promising, safe and non-invasive method for monitoring SA patients [68].

### Periostin

Periostin is a secreted matricellular protein with a key role in amplification and in persistence of chronic inflammation of allergic diseases [81]. Having the ability to bind fibronectin, tenascin-C, collagen I, III and V, periostin is involved in the process of subepithelial fibrosis in asthma patients [82]. Furthermore, it exerts its biological activity also by binding integrins on cell surfaces and activating intracellular signal pathways [83]. Periostin is induced by IL-4 and IL-13 in bronchial epithelial cells and in lung fibroblasts and its expression is correlated with the RBM thickness [82, 84, 85]. Moreover, this molecule is able to accelerate eosinophils tissue infiltration facilitating their adhesion to extracellular matrix proteins [86]. Serum periostin can be considered a systemic biomarker Th2-high asthma related because it is a signature molecule associated to higher AHR, serum IgE, eosinophilic inflammation, subepithelial fibrosis, compared to Th2-low asthma. It is possible to consider serum periostin a promising biomarker for two main reasons. First of all, this protein easily moves from inflamed tissues to blood circulation so its serum concentrations reflects its local production in lesions induced by Th2-type immune responses [87, 88]. Moreover, its basal serum levels are physiologically relatively low (~50 ng/ml) compared to other extracellular matrix proteins such as fibronectin or vitronectin. Jia et al. in the Bronchoscopic Exploratory Research Study of Biomarkers in Corticosteroid-refractory Asthma (BOBCAT), identified serum periostin as the single best systemic biomarker of airway luminal and tissue eosinophilia in severe, uncontrolled asthmatics. Adopting 25 ng/ml serum periostin as an arbitrary cut-off, eosinophil-low and eosinophil-high patients are effectively differentiated, with a positive predicted value of 93 %. This study evidenced the superiority of serum periostin for predicting sputum and tissue eosinophilia, compared to blood eosinophils, IgE levels, YKL-40 and FeNO [89]. More recently, Kanemitsu et al., in an observational study, found that high serum periostin concentration (≥95 ng/ml) is the unique biomarker, among several serum markers, associated with the greater annual decline in FEV1 (at least 30 ml/year) [90]. In addition to being an encouraging biomarker in predicting responders to traditional ICS therapy, periostin seems to also identify responders to new target treatments [91]. Corren et al. demonstrated that its higher serum levels might predict the response to Th2 target therapy with biologic agents such as anti-IL-13 monoclonal antibody (lebrikizumab). The Authors performed a randomized, double-blind, placebo-controlled study of lebrikizumab in 219 adults with unstable asthma despite ICS treatment. The therapy was associated with increased FEV1 values in patients with high pretreatment levels of serum periostin [92]. Similarly, analyzing the results from the EXTRA study performed with uncontrolled, severe, allergic asthmatics, Hanania et al. found that the high serum periostin group had a greater decreased exacerbation rate after omalizumab treatment compared to low serum periostin group [47]. Finally, in a recently published study, Bobolea et al. investigated the potential role of sputum periostin, more organ-targeted than serum periostin, as a biomarker of SA. The Authors found that sputum periostin levels are associated with persistent airflow limitation and eosinophilic inflammatory phenotype despite high-dose ICS therapy [93].

Taken together, these results show that periostin can be a useful biomarker to apply stratified medicine for SA and to yield better outcomes in asthma management. More evaluations are required to validate and clarify the potential utility of periostin in research and before this measurement can be applied in everyday clinical practice. Future studies should better evaluate this biomarker because it was demonstrated that its high levels could be detected in several conditions associated with increased cellular proliferation, angiogenesis, stress, tissues injury not necessarily dependent on a Th2 immune response, as shown in Table 2. We should also remember that periostin is not specific to asthma or the airway epithelium [81, 109, 123, 124]. These considerations should lead clinicians to use an integrative approach which links clinical features and molecular mechanisms and should encourage to better investigate analysis of sputum periostin. It will also be necessary to establish and validate cut-off values to define high and low periostin levels. Furthermore, additional carefully designed studies are needed to evaluate if periostin, alone or in combination with other more conventional biomarkers, can be utilized in better redefining current asthma phenotypes and selecting patients for emerging asthma therapeutics targeting Th2 inflammation.

**Table 2:** Overview on periostin as biomarker in different diseases

	References
*Allergic and respiratory diseases*	
Asthma	[89]
Atopic dermatitis	[94]
IgG4-related sclerosing sialadenitis	[95]
Allergic rhinitis and chronic rhinosinusitis	[96]
Eosinophilic otitis media	[97]
Idiopathic interstitial pneumonias	[98]
Pulmonary fibrosis	[99]
Nasal polyps associated with aspirin-sensitive asthma	[100]
*Oncology*	
Cholangiocarcinoma	[101, 102]
Ovarian carcinoma	[103, 104]
Colon cancer	[105]
Pancreatic cancer	[106, 107]
Melanoma	[108]
Head and neck cancer	[109]
Glioblastoma	[110]
Breast cancer	[111]
Non-small cell lung carcinoma	[112]
*Osteology*	
Bone marrow fibrosis	[113]
Fibrous dysplasia	[114]
*Other inflammatory diseases*	
Systemic sclerosis	[115]
Proliferative diabetic retinopathy	[116]
Psoriasis	[117]
Interstitial renal fibrosis	[118]
Polycystic kidney disease	[119]
Lupus nephritis	[120]
Eosinophilic esophagitis	[86]
Hepatic fibrosis	[121]
Myocardial fibrosis	[122]

### Galectins

**Fig. 2:**
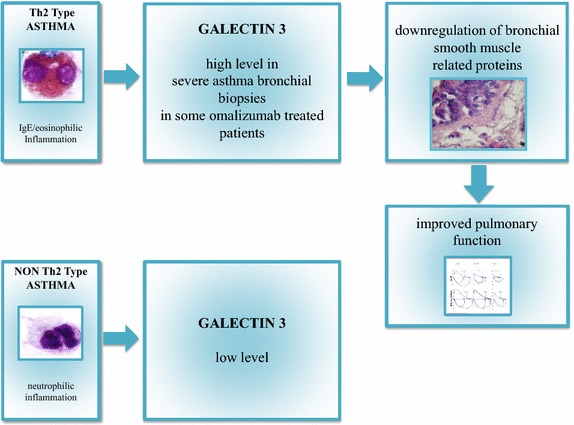
Possible clinical meaning of Galectin 3 in severe asthma.

Galectins are a family of animal lectins with different cellular and extracellular localizations. These proteins bind the cell-surface and extracellular matrix (ECM) glycans and, thereby, affect a variety of cellular processes and biological activities. To date, 15 galectins having a role in physiological and pathobiological processes like cancer, heart failure, tissue repair and platelets aggregation have been found in mammals [125–128]. Regarding airway diseases, the effects of these proteins on the inflammatory process were analyzed for the first time in murine models. Galectin-9 role is still unclear because it was identified as a possible recruiter of eosinophil granulocytes and promoter of Th2 dominance [129], but also as a IgE binding protein with anti-allergic effects able to prevent acute asthma exacerbations [130, 131]. Another galectin with a demonstrated role in asthma is Galectin-3, the only chimera galectin found in Vertebrates, with biological activities in numerous cellular functions like cellular adhesion, growth, chemoattraction, differentiation, apoptosis and cellular cycle as well as an IgE binding protein activity [132–134]. Evaluation of Galectin-3 expression in deficient and wild-type *gal*-*3* mice with OVA-induced asthma, evidenced that OVA-sensitized *gal*-*3* (−/−) mice developed fewer eosinophils, lower goblet cell metaplasia and significantly less AHR after airway OVA challenge compared to similarly treated *gal*-*3* (+/+) mice [135]. Moreover, studies on Gal-3 gene therapy confirmed how it is possible to reduce eosinophils airway infiltration, AHR and tissue remodeling [136]. The involvement of Galectin-3 in human airways inflammatory process has been ascertained for COPD [137], lung fibrosis [138] and asthma. In asthma, Gal-3 expression seems to be related to the development of a specific inflammatory pattern and biological therapy outcome. Recently, Gao et al. found a significantly reduced sputum Gal-3 in patients with neutrophilic asthma [139]. Moreover, evaluating bronchial biopsies of SA patients treated with omalizumab using proteomic technique, we observed that proteomic profile of bronchial tissue before omalizumab treatment presents a typical pattern indicative of anti-IgE treatment response. Galectin-3 was expressed only in subjects with a positive bronchial morphometric analysis response to anti-IgE treatment. In our opinion Galectin-3, having the ability to bind IgE proteins, can be considered a reliable biomarker to predict the modulation of airway remodeling and the improvement of pulmonary function in SA patients before they begin omalizumab therapy (Fig. 2) [37].

## Conclusions

The need in finding biomarkers useful to monitor treatment response is evident in clinical practice, however their discovery is made difficulty by the huge number of proteins involved in severe asthma pathogenesis, only a part of which has been cited in this work. Recently, advances have been obtained by data analysis from genomic and proteomic profiling studies but the application of these methods in clinical practice is difficult. One of the main problems is the cost of many techniques, which require specific instrumentation and skills not easy to achieve. Moreover, protein concentrations may change depending on the inflammatory condition of the patient, disease-associated processes and the sample collecting/analysis method. Nonetheless, each of candidate biomarkers is involved in different biological aspects and gives us information that can be largely overlapping. All these reasons make clear that the road to the identification and the daily use of defined biomarkers in SA is still long and winding. Development of novel serum/sputum-based biomarker panels with improved sensitivity and specificity over the ones currently available, will lead to promising future in the diagnosis of SA [140–142]. Accordingly with Gustafson et al., the more suitable reality in clinical practice will be: a definition of different panels composed by different biomarkers leading to the eligibility of the patients to a certain therapeutic treatment [143].

## Abbreviations

SA: severe asthma
ICS: inhaled corticosteroids
ERS: European Respiratory Society
ATS: American Thoracic Society
FEV1: forced expiratory volume in 1 second
FVC: forced vital capacity
Th: T helper lymphocyte
AHR: airway hyperresponsiveness
NADPH: nicotinamide adenine dinucleotide phosphate oxidase
ROS: reactive oxygen species
RAGE: receptor for advanced glycation end-products
sRAGE: soluble form of receptor for advanced glycation end-products
COPD: chronic obstructive pulmonary disease
ADAM8: metalloproteinase domain 8
TGF-β: trasforming growth factor β
RBM: reticular basement membrane
FeNO: fractional exhaled nitric oxide
IgE: immunoglobulin E
IL: interleukin
CCL11: eotaxin-1
BALF: bronchoalveolar lavage fluid
EBC: exhaled breath condensate
NOS: nitric oxide synthase
NO: nitric oxide
EB: exhaled breath
LTB4: leukotriene B4
LXA4: lipoxin A4
ECM: extracellular matrix
